# Suspected Acute Disseminated Encephalomyelitis Presenting With Persistent Vegetative State and Full Neurological Recovery in an Adult: A Case Study

**DOI:** 10.7759/cureus.111203

**Published:** 2026-06-20

**Authors:** Jonah Im, Mahmoud Hamza, Sylvia Silver, Stuart D Harper, Kassi Kronfeld

**Affiliations:** 1 Medicine, University of California Los Angeles David Geffen School of Medicine, Los Angeles, USA; 2 Neurology, Harbor University of California Los Angeles Medical Center, Los Angeles, USA; 3 College of Letters and Sciences, University of California, Los Angeles, Los Angeles, USA

**Keywords:** acute disseminated encephalomyelitis (adem), autoimmune encephalitis, encephalopathy, posterior reversible encephalopathy syndrome (pres), vegetative state

## Abstract

Acute disseminated encephalomyelitis (ADEM) is an autoimmune inflammatory disease of the central nervous system that typically presents with headache, fever, altered consciousness, and seizures. Diagnosis is made clinically and radiologically, supported by the exclusion of mimics. Delayed treatment can lead to severe neurological impairment, including a persistent vegetative state. A 69-year-old Asian female with rheumatic heart disease and prior transverse myelitis presented with hypoxic respiratory failure. On hospital day (HD) 4, she developed a rapid decline in mental status, progressing to a vegetative state with electroclinical seizures. Brain MRI showed diffuse, symmetric T2/fluid-attenuated inversion recovery (FLAIR) hyperintensities involving subcortical white matter, which is an atypical finding for classic ADEM. Extensive infectious and autoimmune testing was unrevealing. Lumbar puncture on HD 11 (prior to steroids) showed no pleocytosis. She remained vegetative for 39 days without response to high-dose corticosteroids. Following a five-day course of intravenous immunoglobulin (IVIG), she showed marked neurological recovery. At discharge, she returned to her baseline functional status. This case illustrates the diagnostic challenges of suspected ADEM in an older adult with atypical symmetric imaging, prolonged vegetative state, and delayed response to immunotherapy. The case adds to the limited literature on extended encephalopathy in suspected ADEM and underscores the role of IVIG when steroid response is unclear.

## Introduction

Acute disseminated encephalomyelitis (ADEM) is an acute neurological condition that causes demyelination of the central nervous system. The annual incidence is estimated to be 0.4-0.8 per 100,000, with the mean age at presentation between six and eight years old [1]. Numerous cases are reported in adults and elderly patients; however, the incidence is significantly lower [2]. Although the exact mechanism of ADEM is not fully understood, it typically precedes an infection or immunization, which is thought to lead to unregulated neuroinflammation in genetically susceptible individuals. An antibody or cell-mediated response to this immune trigger is believed to react with myelin-associated proteins, leading to an acute, widespread demyelinating event [3-5].

On T2-weighted MRI and fluid-attenuated inversion recovery (FLAIR), ADEM is associated with large, multifocal, and hyperintense lesions commonly found in the cerebral white matter; lesions may also occur in the brainstem, cerebellum, and spinal cord [3,6]. Cerebrospinal fluid analysis is generally normal but may show mild protein elevation with lymphocytic pleocytosis, though it is not a primary diagnostic tool [1].

Symptoms typically manifest abruptly within six days to six weeks of an infectious trigger, with headache, fever, nausea, vomiting, and fatigue being the earliest signs, which can progress to seizures, acute encephalopathy, focal neurological deficits, and coma within days if left untreated [1,3,7]. With timely treatment, including first-line IV methylprednisolone or second-line intravenous immunoglobulin (IVIG), clinical symptoms and MRI findings typically begin to resolve within a few days after treatment [1,8,9].

## Case presentation

A 69-year-old Asian woman with a history of rheumatic heart disease status post mechanical mitral valve replacement on warfarin, severe aortic stenosis, atrial fibrillation with rapid ventricular response, reported transverse myelitis with baseline paraplegia, a chronic Foley catheter, and a prior right cerebellar infarct presented from a skilled nursing facility with acute hypoxic respiratory failure. Her baseline modified Rankin scale (mRS) was 4 due to prior transverse myelitis with paraplegia [10]. The Glasgow Coma Scale (GCS) at baseline was 15. Her hypoxia resolved quickly with ventilatory support, and she returned to her baseline mental status without an intervening lucid interval. Her hospital course was complicated by methicillin-resistant *Staphylococcus aureus* pneumonia treated with vancomycin, *Pseudomonas* bacteremia, transaminitis, and a type 2 non-ST-elevation myocardial infarction (NSTEMI).

On hospital day (HD) 4, she developed an abrupt decline in mental status. She was previously alert and oriented and became unresponsive within 24 hours, showing only eye opening to voice without tracking, withdrawal to pain, or spontaneous movement. GCS declined to a nadir of 5 (E1, V1, M3).

Continuous EEG monitoring was initiated on HD 5 (Figure 1). The background demonstrated moderate slowing with poor organization and absent posterior dominant rhythm, indicative of moderate-to-severe diffuse or multifocal cerebral dysfunction. There were continuous left lateralized periodic discharges (LPDs) at approximately 1.5-2 Hz. One electroclinical seizure was captured at approximately 11:36, originating as spike waves at O1 and evolving to polyspike waves over approximately three minutes, accompanied by head turning to the right. The seizure was followed by diffuse background attenuation. These findings did not meet the Salzburg criteria for nonconvulsive status epilepticus (NCSE) [11]. The patient was intubated for airway protection and transferred to the neuro ICU. Generalized periodic discharges (GPDs) ranging from 0.5 to 2 Hz were initially noted on bedside review. Antiseizure medications were initiated (lacosamide 250 mg twice daily, levetiracetam 2 g twice daily, and clobazam 15 mg twice daily), and periodic discharges eventually resolved. Antiseizure medications were slowly weaned to levetiracetam monotherapy by discharge.

**Figure 1:**
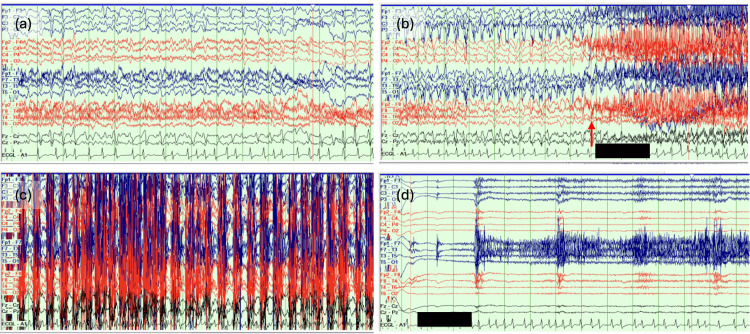
Serial EEG recordings obtained during hospital day 5 illustrating the evolution of epileptiform activity. A: left hemispheric lateralized periodic discharges (LPDs); B: evolution of LPDs into an electrographic seizure (the red arrow denotes seizure onset); C: electrographic seizure at mid-event; D: post-ictal diffuse background attenuation following seizure termination.

MRI of the brain demonstrated extensive confluent T2/FLAIR hyperintensities in the supratentorial and infratentorial subcortical white matter without enhancement (Figure 2). The distribution was symmetric and diffuse, which is atypical for classic ADEM. Differential diagnosis included ADEM, posterior reversible encephalopathy syndrome (PRES), reversible cerebral vasoconstriction syndrome, vasculitis, or other leukoencephalopathies. Cervical and thoracic spine MRI showed myelomalacia without acute demyelinating lesions (Figure 3).

**Figure 2:**
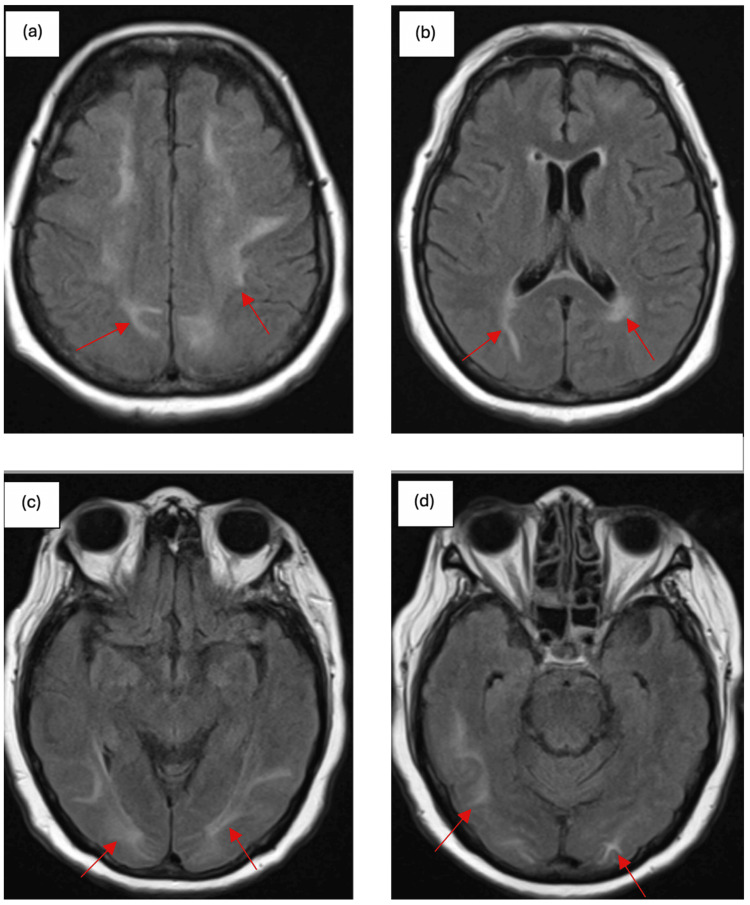
Initial axial brain MRI T2-weighted images (hospital day 14) demonstrating confluent hyperintensities in the deep white matter of the occipital and parietal lobes. A-D: represent sequential axial levels from superior to inferior (red arrows indicate areas of hyperintensity).

**Figure 3:**
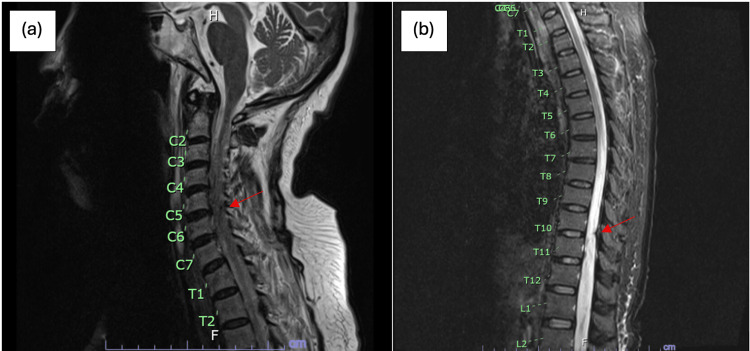
Cervical and thoracic spine MRI (hospital day 5). A: MRI imaging of the cervical spine; B: MRI imaging of the thoracic spine (red arrows indicate areas of myelomalacia). No acute demyelinating lesions were appreciated on imaging.

PRES was considered in the differential diagnosis. Blood pressure trends during hospitalization ranged from systolic 80s to 160s mmHg, with a single reading exceeding 160 mmHg systolic and no diastolic reading above 100 mmHg. No antihypertensive treatment was administered. Renal function was stable (creatinine 1.12 mg/dL at peak, no baseline available); no dialysis was required. The patient had no exposure to calcineurin inhibitors, chemotherapy, or other immunosuppressants before admission. While PRES could not be definitively excluded given the multifocal T2/FLAIR hyperintensities, the clinical course was atypical for PRES: the patient remained vegetative for 39 days without rapid improvement following blood pressure normalization, and there was no identifiable acute trigger (e.g., hypertensive crisis, eclampsia, or toxin exposure). However, the authors acknowledge that PRES remains a diagnostic consideration that cannot be fully excluded.

Lumbar puncture was performed on HD 11, which was before initiation of high-dose intravenous methylprednisolone (HDs 18-22). Therefore, CSF findings were not altered by corticosteroid therapy. Laboratory results include no pleocytosis, elevated glucose and protein, negative oligoclonal bands, and a normal IgG index, negative cytology, and negative broad infectious testing (Table 1). Serum autoimmune workup was notable for a positive ANA; all other antibodies were negative. A subsequent image-guided lumbar puncture performed later in the hospitalization (after IVIG) similarly showed no diagnostic abnormalities (Table 1).

**Table 1:** Laboratory investigations during hospitalization. Reference ranges per institution standard. Directionality indicates deviation from the reference range. "—" denotes not tested at that time point. HD: hospital day; WBC: white blood cell; IgG: immunoglobulin G; MOG: myelin oligodendrocyte glycoprotein; AQP4: aquaporin-4; ANA: antinuclear antibody; SSA/SSB: Sjogren’s-syndrome-related antigen A/B; C-ANCA/P-ANCA: cytoplasmic/perinuclear anti-neutrophil cytoplasmic antibody; anti-MPO: anti-myeloperoxidase; MuSK: muscle-specific kinase; SPEP: serum protein electrophoresis; ESR: erythrocyte sedimentation rate; CRP: C-reactive protein; C3/C4: complement components 3 and 4; CSF: cerebrospinal fluid; LP: lumbar puncture; IVIG: intravenous immunoglobulin; HSV: herpes simplex virus; HHV: human herpesvirus; CMV: cytomegalovirus; EBV: Epstein-Barr virus; VZV: varicella-zoster virus; HIV: human immunodeficiency virus; VDRL: venereal disease research laboratory; MTB: *Mycobacterium tuberculosis*; PCR: polymerase chain reaction

Parameter	Reference range	HD 11 (pre-steroids)	HD 15	Post-IVIG
CSF Studies (LP - HD 11)				
WBC	0-5 cells/μL	Normal, 1 cell	—	Normal, 1 cell
Glucose	45-80 mg/dL	Elevated, 89 mg/dL	—	—
Protein	15-45 mg/dL	Elevated, 58 mg/dL	—	—
Oligoclonal bands	Negative	Negative	—	—
IgG index	0.28-0.66	Normal, 0.34	—	—
Cytology	Negative	Negative	—	—
Infectious panel: HSV-1/2, HHV-6, CMV, EBV, VZV, HIV, enterovirus, parechovirus, Cryptococcus, Coccidioides, Lyme, VDRL, bacteria, fungi, MTB PCR	Negative	All negative	—	—
Serum Autoimmune and Inflammatory Markers				
MOG-IgG	Negative	Negative	—	—
AQP4-IgG	Negative	Negative	—	—
ANA	Negative <1:40	Positive 1:160, nucleolar pattern	—	—
SSA / SSB	Negative	Negative	—	—
C-ANCA / P-ANCA / anti-MPO	Negative	Negative	—	—
Anti-MuSK	Negative	Negative	—	—
Cryoglobulins	Negative	Negative	—	—
SPEP	Negative	Negative	—	—
ESR	0-20 mm/hr (F)	—	Elevated, 80 mm/hr	—
CRP	<10 mg/L	—	Elevated, 22 mg/L	—
C3	90-180 mg/dL	—	Low, 45 mg/dL	—
C4	16-47 mg/dL	—	Low, 11 mg/dL	—

Given diagnostic uncertainty, she was started on pulse-dose methylprednisolone 1 g/day from HD 16-20, followed by a prednisone taper beginning at 1 mg/kg/day (70 mg) from HD 21-34, then 0.85 mg/kg/day (60 mg) from HD 35-41, and 0.7 mg/kg/day (50 mg) from HD 42-45. Despite partial radiographic improvement (Figure 4), she remained clinically vegetative.

**Figure 4:**
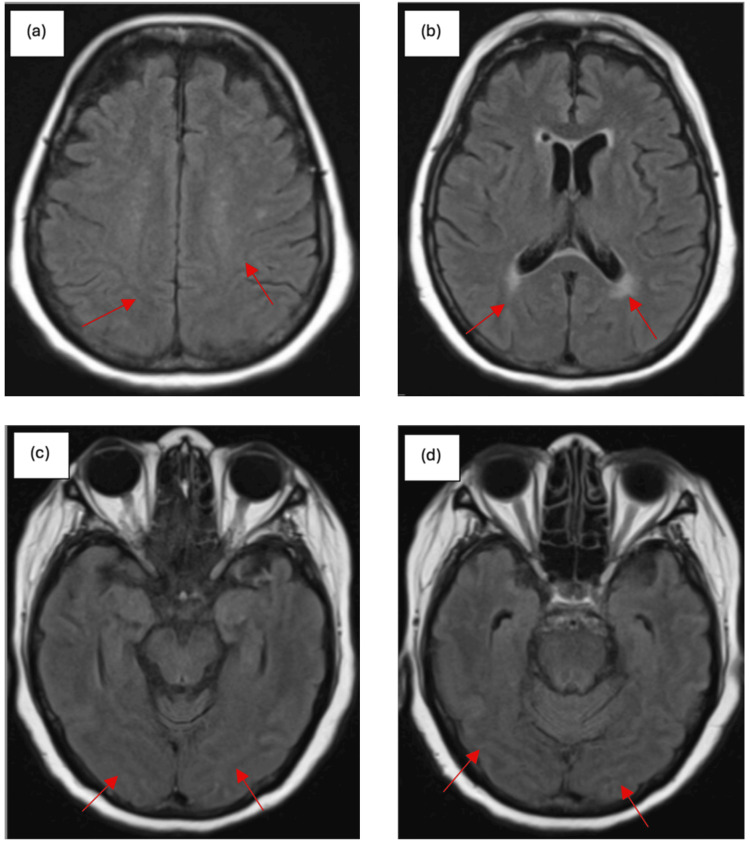
Follow-up axial MRI T2-weighted image (hospital day 37) showing complete resolution of hyperintensities demonstrated in Figure 1. A-D: correspond to the same axial levels shown in Figure 1, with red arrows indicating the prior locations of these hyperintensities.

Given the lack of steroid response, she received IVIG 0.4 g/kg/day from HD 39-43 for suspected ADEM. On HD 43 (fifth day of IVIG), she showed significant neurological improvement, including command following, purposeful upper extremity movement, and orientation to person, place, and time. She continued a prednisone taper. She required tracheostomy for prolonged mechanical ventilation but was gradually weaned, passed swallow evaluation, and had tracheostomy removed on HD 92.

At discharge, the patient had returned to her baseline functional status (mRS 4) with GCS 15. Formal cognitive testing (e.g., Montreal Cognitive Assessment (MoCA) or Mini-Mental State Examination (MMSE)) was not performed, but family and nursing staff confirmed return to premorbid orientation and daily function. At three-month follow-up, she remained at a skilled nursing facility with no persistent neurological deficits beyond her prior baseline paraplegia (Figure 5). The patient reported no memory of the acute vegetative phase and expressed gratitude to the medical team.

**Figure 5:**
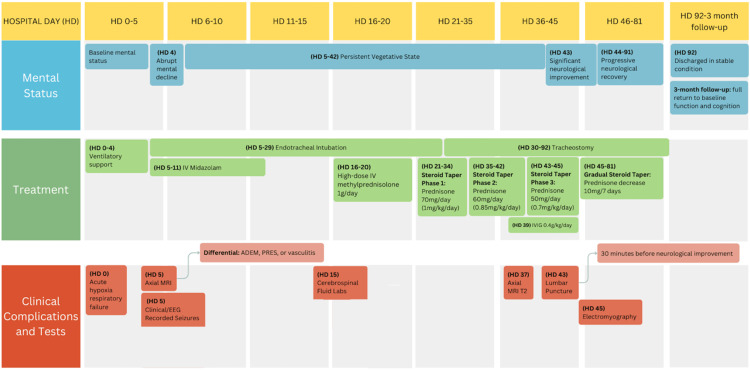
Timeline of hospital stay, organized by mental status, treatment course, and clinical complications and tests. PRES: posterior reversible encephalopathy syndrome; ADEM: acute disseminated encephalomyelitis; HD: hospital day The figure was created using Canva (Canva Pty Ltd, Sydney, Australia).

## Discussion

This case describes an older adult with suspected ADEM who developed a prolonged vegetative state lasting 39 days, followed by full neurological recovery after IVIG therapy. The case is notable for several atypical features that complicated diagnosis: symmetric and confluent T2/FLAIR hyperintensities (uncommon in ADEM), absence of CSF pleocytosis, and lack of early response to high-dose corticosteroids. 

ADEM was initially suspected due to the patient's recent respiratory infection and history of transverse myelitis, suggesting an underlying immune predisposition. However, symmetric white matter involvement is atypical for ADEM, which classically produces large, multifocal, asymmetric lesions [12,13]. This broadened the differential to include PRES, vasculitis, and other leukoencephalopathies.

PRES remained a diagnostic consideration throughout. While the patient had only a single mild BP elevation (systolic 160s) without hypertensive crisis, PRES has been reported in normotensive patients [14,15] and can be triggered by sepsis or systemic inflammation [16,17]. However, the 39-day vegetative state without rapid improvement following BP normalization argues against typical PRES, which usually stabilizes within days [17,18]. The absence of an acute trigger (eclampsia, renal failure, or calcineurin inhibitors) further reduced, though did not eliminate, the likelihood of PRES.

The lack of comprehensive autoimmune encephalitis testing is a significant limitation. NMDA-R antibody encephalitis, LGI1-antibody encephalitis, and paraneoplastic syndromes can present with altered mental status, seizures, and prolonged encephalopathy. These diagnoses cannot be excluded in this case. However, the absence of typical features (facial-brachial dystonic seizures in LGI1 or orofacial dyskinesias in NMDA-R), negative CSF cytology, and absence of malignancy make them less likely.

Steroid-resistant ADEM is uncommon, with reported rates of approximately 16% [19]. Age-related changes in blood-brain barrier integrity and glucocorticoid receptor function may have contributed to the lack of early response in this patient. The delayed improvement following IVIG supports an immune-mediated process and underscores the importance of early consideration of second-line therapy when corticosteroids fail to produce timely clinical change.

Prolonged vegetative state in ADEM is rare, particularly in adults. Prior case reports describe widely varying outcomes, including persistent vegetative state despite aggressive immunotherapy [20] as well as full recovery after several months [21,22]. Our case adds to this small body of literature by documenting full recovery after 39 days of vegetative state in an older adult, which is an outcome that may help guide prognostic discussions in similar cases.

This case report has several limitations. First, a full autoimmune encephalitis and paraneoplastic panel, including NMDA receptor antibody (serum/CSF), LGI1, CASPR2, GABA-B, AMPA, and Hu/Yo/Ri/Ma2/CV2, was not performed during the acute hospitalization. This limits the ability to definitively exclude autoimmune encephalitis as an alternative diagnosis. Second, no formal cognitive testing (e.g., MoCA or MMSE) was administered at follow-up; functional recovery was assessed clinically and by family report. Third, while the patient had only a single elevated blood pressure reading (systolic 160s) without hypertensive crisis, PRES cannot be fully excluded as an alternative diagnosis. Despite these limitations, the acute monophasic presentation, negative infectious and autoimmune workup (to the extent performed), and eventual response to immunotherapy most strongly supported suspected ADEM as the working diagnosis.

## Conclusions

This case illustrates the diagnostic difficulties of suspected ADEM in older adults with atypical imaging, a prolonged vegetative state, and a delayed treatment response. It highlights the importance of systematically excluding PRES and autoimmune encephalitis, acknowledging diagnostic uncertainty, and considering IVIG when corticosteroids do not lead to timely improvement. The favorable outcome despite prolonged encephalopathy offers prognostic reassurance in similar challenging cases.
